# Two novel *SUCLA2* variants cause mitochondrial DNA depletion syndrome, type 5 in two siblings

**DOI:** 10.3389/fneur.2024.1394150

**Published:** 2024-07-11

**Authors:** Xiaohuan Zhang, Guo Zhang, Li Cao, Wenjing Zhou, Chang Tan, Shi Ma, Jiyun Yang

**Affiliations:** ^1^Sichuan Provincial Key Laboratory for Human Disease Gene Study, Center of Medical Genetics, Sichuan Provincial People’s Hospital, School of Medicine, University of Electronic Science and Technology of China, Chengdu, China; ^2^Research Unit for Blindness Prevention of Chinese Academy of Medical Sciences (2019RU026), Sichuan Academy of Medical Sciences, Chengdu, China; ^3^Dean’s Office, Sichuan Provincial People’s Hospital, School of Medicine, University of Electronic Science and Technology of China, Chengdu, China

**Keywords:** MTDPS-5, *SUCLA2*, mitochondrial succinate-CoA ligase, NGS, mitochondrial DNA, rare disease

## Abstract

Mitochondrial DNA depletion syndrome (MDS), characterized by succinate-CoA ligase deficiency and loss of mitochondrial DNA (mtDNA), is caused by specific variants in nuclear genes responsible for mtDNA maintenance. *SUCLA2*-related mitochondrial DNA depletion syndrome, type 5 (MTDPS-5), presents as a rare, severe early progressive encephalomyopathy. This report investigates a new family exhibiting clinical manifestations of MTDPS-5 and elucidates the genetic basis of this disorder. In two affected siblings, a novel maternally inherited nonsense variant [c.1234C>T (p.Arg412*)] in the *SUCLA2* gene and a unique paternally inherited indel variant (g.48569263–48571020del1758insATGA) were identified. Additionally, the siblings exhibited blood mtDNA content lower than 33% compared to age-matched controls. These findings underscore the importance of assessing *SUCLA2* variants in patients with severe early progressive encephalomyopathy, even in the absence of methylmalonic aciduria or mtDNA loss, thereby broaden the mutational spectrum of this gene.

## 1 Introduction

Mitochondrial DNA depletion syndrome (MDS) includes various disorders characterized by genetic and clinical diversity, marked by a reduction in affected tissues mitochondrial DNA (mtDNA) copies, leading to diminished synthesis of mitochondrial respiratory chain complexes (1). Nuclear genes implicated in MDS include *TK2*, *DGUOK*, *RRM2B*, *POLG*, *MPV17*, *MGME1*, *C10orf2*, *FBXL4*, *SUCLA2* and *SUCLG1* (2, 3).

Pathogenic variants in *SUCLA2* have been associated with mitochondrial DNA depletion syndrome, type 5 (MTDPS-5, OMIM ID: 612073) (4). The *SUCLA2* gene is approximately 60 kb in length and is responsible for encoding 463 amino acids within the β-subunit of the ADP-forming succinyl-CoA ligase (SUCL-A), a key Krebs cycle enzyme. In the citric acid cycle, this enzyme plays a crucial role in the reversible conversion of succinyl-CoA to succinate. Succinyl-CoA synthase disrupts its tight interaction with mitochondrial nucleoside diphosphate kinase, thereby compromising mitochondrial DNA synthesis and leading to depletion of mitochondrial DNA. It also stabilizes mitochondrial nucleotide diphosphokinase (NDPK), involved in the dNTP salvage pathway in mtDNA replication (5, 6). *SUCLA2* protein is primarily expressed in cardiac, skeletal muscle, and cerebral tissues (7, 8). Pathogenic variants in *SUCLA2* lead to MTDPS-5 disease characterized by neurosensory deafness, early hypotonia, severe developmental delay, progressive neurological decline, muscle contractures, and dystonia/athetosis. These variants often result in mild increases in plasma lactate, elevations in plasma carnitine esters and methylmalonic acid, and urinary excretion of methylmalonic acid. Cerebral imaging reveals anomalies in the basal ganglia (e.g., caudate and putamen nuclei) and occasional demyelination (4, 9–16). Nowadays, Next-Generation Sequencing (NGS) allows cost-effective and timely assessment of the human genome in small families or individuals, facilitating the study of genotype-phenotype interactions in genetically and clinically diverse diseases such as mitochondrial dysfunction.

In this case, we present a Chinese family exhibiting a rare and severe early progressive encephalomyopathy, diagnosed with mitochondrial DNA depletion syndrome, type 5 (MTDPS-5), and carrying compound heterozygous variants (c.1234C>T; g.48569263–48571020del1758insATGA) in the *SUCLA2* gene. The predominant symptoms observed in most patients included hypotonia, severe encephalopathy, generalized dystonia, muscle contractures, feeding difficulties, lack of voluntary movement, deafness as well as elevated levels of lactate and methylmalonic acid in plasma. Our manuscript aligns with the focus of the selected journal and its specific field.

## 2 Case report

### 2.1 Clinical description

**Figure 1:**
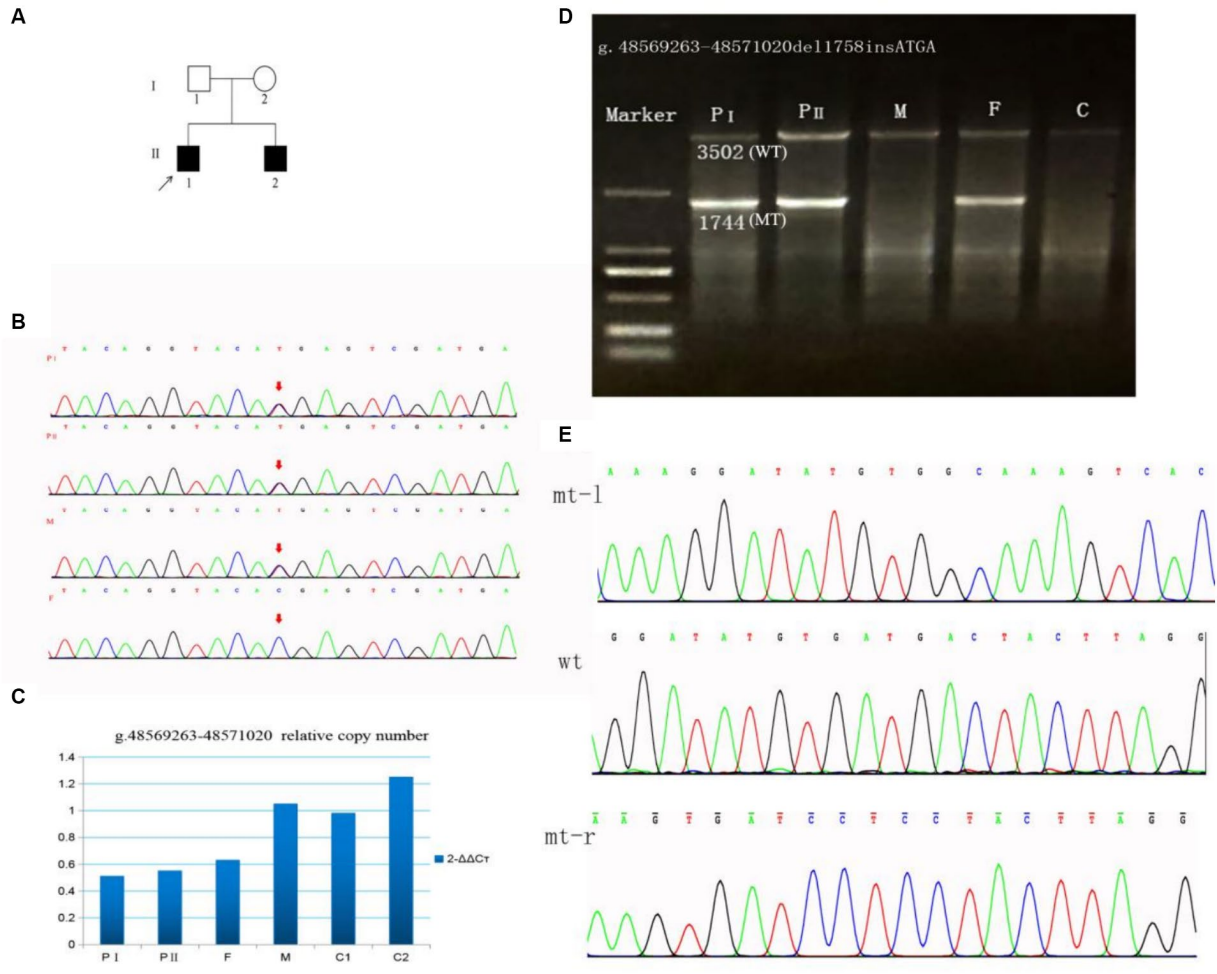
A family with *SUCLA2* (NM_003850.2) variation was identified through Sanger sequencing and qPCR. **(A)** Pedigree of the family. **(B)** Sanger sequencing of the c.1234C>T variant in *SUCLA2*. **(C)** g.48569263–48571020del1758 allele copy number. **(D)** Long-range PCR products of the g.48569263–48571020del1758 variant. The 3,502 bases band is the wild-type, and the 1,744 bases band is the mutant. **(E)** Sanger sequencing of the g.48569263–48571020del1758insATGA variant in *SUCLA2*. mt-l, left of breakpoint; wt, wild type; mt-r, right of breakpoint; F, father; M, mother; C, control.

**Figure 2:**
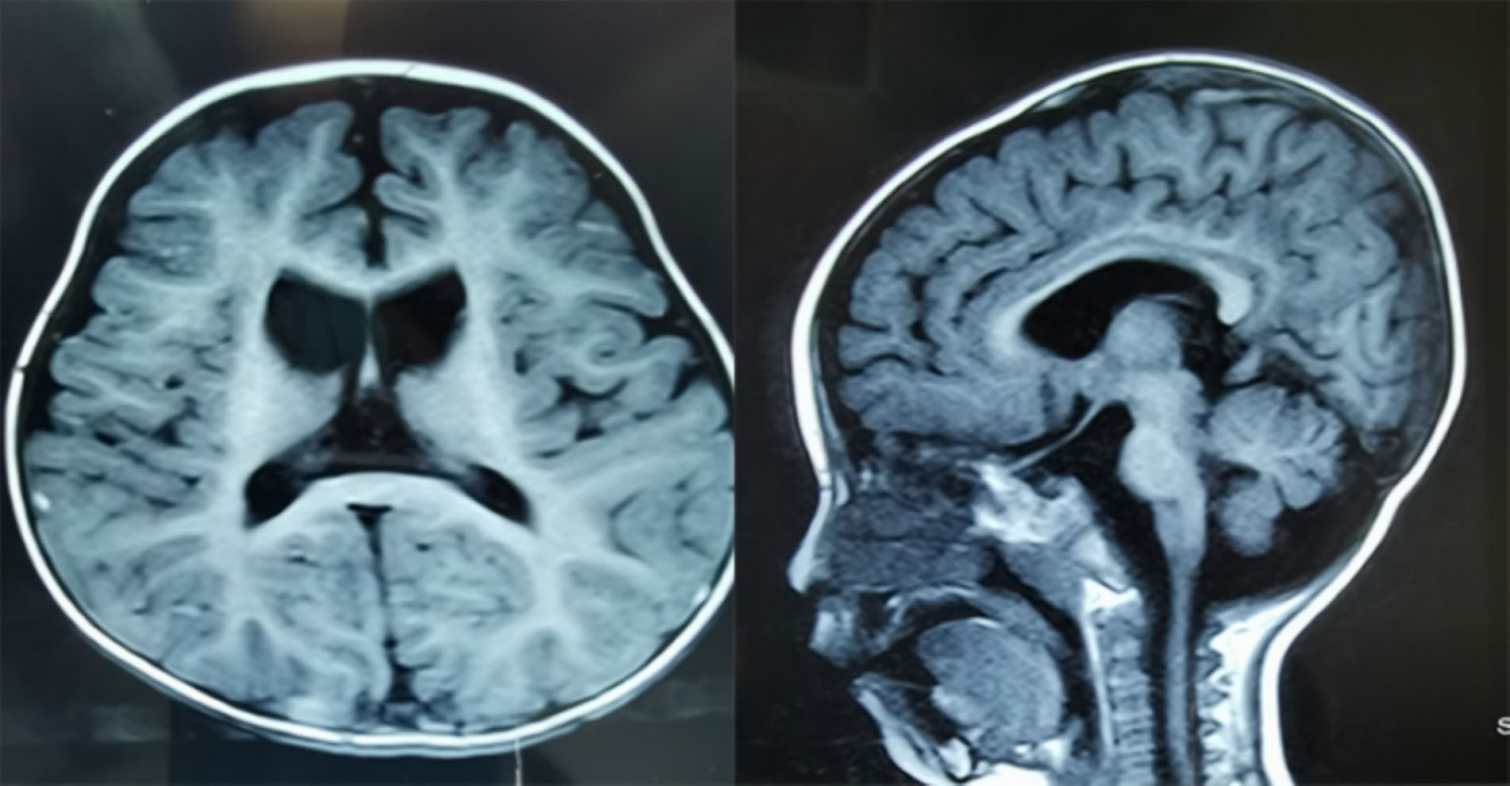
Brain MRI of the PI exhibiting bilateral hyperintensity within the putamina nuclei and caudate (left panel), as well as mild medullary and cerebellar atrophy (right panel).

The proband (PI) was born at full term without asphyxia in Sichuan, China, with a head circumference of 36 cm, birth weight of 2,825 g, and length of 49 cm. There was no family history of neurodegenerative disorders (Figure 1A). Mild hypotonia was noted at birth, which progressed to extreme hypotonia with reduced eye contact and hearing loss by the age of 6 months. Delayed motor milestones and severe deafness were observed by the age of 2 years, rendering the proband unable to walk or sit and experiencing feeding difficulties resulting in poor growth. At the time of our investigation, the proband was 8 years old, presenting with hypotonia, severe encephalopathy, muscle contractures, and generalized dystonia. Axial T2-weighted magnetic resonance imaging (MRI) revealed hyperintensity within the basal ganglia and bilateral cortical atrophy (Figure 2). Laboratory tests indicated slightly elevated blood lactate levels (3.8 mmoL/L; reference: 0.7–2.1). Blood pyruvate and ammonia levels were within normal ranges. Amino acid analysis of blood samples showed increased levels of succinylcarnitine (C4DC) (2.553 μmol/mmol; reference: 0.06–0.45) and acetylcarnitine (34.5 μmol/mmol; reference <27). Urine organic acids analysis revealed elevated levels of methylmalonic acid (MMA) (6.9 μmol/mmol creatinine; reference <4.0) and methylcitric acid (0.8 μmol/mmol creatinine, reference <0.7).

The proband’s younger brother (PII) was born at full term without perinatal asphyxia, with a head circumference of 35 cm, birth weight of 3,017 g, and length of 51 cm. At age of 4 months, he underwent examination which revealed sensorineural hearing loss, limb hypertonia, axial hypotonia, dystonic postures, and brisk tendon reflexes. Laboratory investigations yielded results similar to those of his older brother (Table 1).

**Table 1:** Laboratory finding in the patients described in this study.

	Lactate (in mmol/L) reference 0.7–2.1 mmoL/L	Acetylcarnitine (in μmol/mmol) reference 0–27 μmol/mmol	Succinylcarnitine (in μmol/mmol) reference 0.06–0.45 μmol/mmol	MMA (in μmol/mmol creatinine) reference <4.0 μmol/mmol creatinine	Methylcitric acid (in μmol/mmol creatinine) reference <0.7 μmol/mmol creatinine
PI	3.8	34.5	2.6	6.9	0.8
PII	8.6	47.2	2.7	7.6	0.9

### 2.2 Genetic analysis

WES testing was performed on the proband, revealing a novel heterozygous variant c.1234C>T (p.Arg412*) in exon 10 of the *SUCLA2* gene, which is associated with MTDPS-5. The clinical symptoms observed in the proband closely resemble the phenotype of MTDPS-5. However, no other potentially pathogenic variants or underlying genes related to *SUCLA2* were detected through WES analysis, posing challenges in confirming the diagnosis for this individual. Subsequently, we identified the presence of the variant c.1234C>T (p.Arg412*) through sanger sequencing. Furthermore, a large deletion variant (g.48569263–48571020del1758) in *SUCLA2* gene was detected through WGS analysis. As revealed by sanger sequencing, the c.1234C>T variant identified as maternally inherited (Figure 1B). According to the genomic qPCR analysis, the patients and their father exhibited one copy for the allele at the variant position g.486263–48571020del1758, whereas normal individuals displayed two copies (Figure 1C). To further clarify the breakpoints of this deletion variant, primers for long-range PCR were designed for analysis on both the proband and his family members. The results obtained from long-range PCR showed that amplification products from both brothers and their father exhibited sizes of 3,502 bases and 1744 bases, while amplification product from the mother and control individuals displayed sizes of 3,502 bases (Figure 1D). Subsequently, sanger sequencing of these amplified bands confirmed that this deletion variant was actually an indel variant (g.48569263–48571020del1758insATGA) (Figure 1E).

Our patients carry two novel variants in *SUCLA2*. The variant c.1234C>T (p.Arg412*) is a nonsense variant (PVS1) and has been found in one out of 140,120 individuals in the global control chromosome database (GnomAD), with a carrier frequency of 0.000007 (PM2). The siblings inherited the c.1234C>T variant from their mother and it forms a compound heterozygosity with the paternal variant g.48569263–48571020del1758insATGA (PM3). The clinical symptoms observed in these patients are consistent with the phenotype associated with *SUCLA2* (PP4). According to the ACMG guidelines, this variant is classified as a pathogenic variant (PVS1 + PM2 + PM3 + PP4). The g.48569263–48571020del1758insATGA variant is a large fragment indel variant (PVS1) and it is also a rare variant not exist in global control chromosome database (PM2). Both patients inherited the variant g.48569263–48571020del1758insATGA from their father (PM3). Thus, g.48569263–48571020del1758insATGA was rated as a pathogenic variant in line with ACMG guidelines (PVS1 + PM2 + PM3 + PP4).

**Figure 3:**
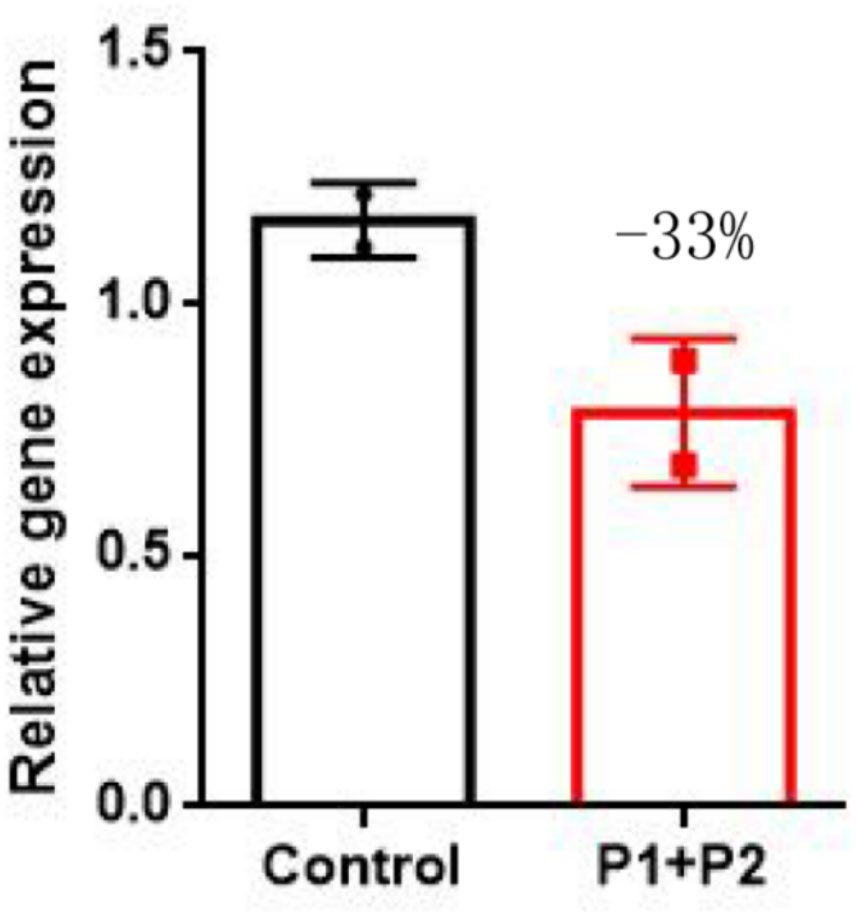
qPCR results revealed a significant decrease in the *SUCLA2* level in two siblings compared with that in age-matched controls. The data are presented as the means ± SEMs of 3 separate assays. One-way ANOVA was carried out for statistical analysis (*p*-value <0.05).

To further investigate the impact of *SUCLA2* variant on cellular mitochondrial content, we employed qPCR to quantify peripheral blood mtDNA levels. Our findings revealed that two siblings had a value lower than 33% compared to the average mtDNA content of age-matched controls (Figure 3). This outcome suggests a partial contribution of these mutations towards mitochondrial depletion in patients’ peripheral blood cells.

## 3 Discussion and conclusion

Variants in *SUCLA2* are exceptionally rare, with only 56 reported cases documented to date. Upon reviewing previous cases, no instances of mild clinical disease presentation were found (Supplementary Table S1). Typically, symptoms in previous MTDPS-5 patients manifest shortly after birth. This study focuses on two Chinese patients from the familial background who are affected by *SUCLA2* (c.1234C>T; g.48569263–48571020del1758insATGA) variants. These patients exhibited symptoms at 4–5 months of age, including hypotonia, severe encephalopathy, generalized dystonia, muscle contractures, feeding difficulties, lack of voluntary movement, and deafness. MRI scans showed hyperintensity within the basal ganglia and bilateral cortical atrophy in these patients. Elevated lactate levels in plasma, along with increased urinary excretion of acetylcarnitine, MMA, and C4DC, were noted in these patients; however, the magnitudes of these metabolic abnormalities was not particularly significant. Furthermore, their symptoms were consistent with those seen in prior patients carrying *SUCLA2* variants associated with TCA cycle defects. Neuromuscular symptoms coupled with a mild elevation of MMA and lactate levels in both plasma and urine, are typically seen in MTDPS-5 patients resulting from such variants. Variants in *SUCLA2* have been reported in a limited number of individuals, primarily originating from the Faroe Islands, where a frameshift variant (c.534 + 1G>A) within intron 4 is prevalent, leading to exon 4 skipping and premature stop codon formation in most individuals (9, 17). Elpeleg et al. (4) initially reported on two Muslim siblings harboring a homozygous indel variant (c.789_802 + 29delinsATAAA). Carrozzo et al. (10) also documented a case of a Turkish individual carrying a homozygous nonsense variant (c.750C>A) within *SUCLA2*. These patients presented severe early-onset encephalopathy accompanied by deafness, with alterations observed within basal ganglia structures as well as feeding difficulties, hypotonia, generalized dystonia, muscle contractures, lack of voluntary movement, and mild MMA. The phenotypes observed in our patients resemble those of individuals with loss-of-function variants (deletions, nonsense, and frameshift variants). However, it is worth noting that pathogenic missense variants may retain some residual enzyme activity. As a result, MTDPS-5 patients harboring distinct missense variants of *SUCLA2* exhibit a comparatively milder phenotype. Furthermore, the survival rate among affected individuals with missense variants surpasses that of those with loss-of-function variants (median survival age 21 years versus 15 years) (9–11, 15, 18).

Variants in *SUCLA2* genes related to mtDNA synthesis may impact the mtDNA content. Although muscle mtDNA content remained consistent, the parents declined invasive diagnostic procedures. Previous studies have utilized blood-extracted DNA to assess mtDNA levels in individuals with *SUCLA2* gene variants (19). However, given the age-related decline in blood mtDNA content, we employed blood samples from age-matched healthy children as controls (20). Moreover, our results revealed a notable reduction in mtDNA levels in the affected siblings compared to controls of similar age. Blood samples have specificity but lack sensitivity in detecting mtDNA depletion. Therefore, blood mtDNA content evaluation is only indicative but not diagnostic. Following on the ACMG guidelines, these variants were identified as disease-causing. The indel variant g.48569263–48571020del1758insATGA is located at the exon 2-IVS2 junction of the *SUCLA2* gene and may lead to cDNA truncation, resulting in a potential truncation of 77–89 amino acids. Since the ATP-grasp domain is created by amino acids 61–288, with 77–89 amino acids located within the domain, truncation of the peptide chains may lead to changes in protein structure. The c.1234C>T variant may result in the premature stop codon which can be also a consequence of non sense mediated mRNA decay (NMD). However, structural modeling based on a template protein structure suggested also that the lack of this stretch may induce a conformational change (Supplementary Figure S1). A comparison of the wild type of *SUCLA2* protein structure (WT), g.48569263–48571020del1758insATGA (MT-1) variant demonstrated a significant transformation from an α-helix and β-fold to an elongated loop (highlighted in red). The c.1234C>T (MT-2) variant exhibited the loss of two α-helices and β-fold at the protein’s C-terminal (highlighted in black), while an additional α-helix appears at amino acid positions 301–307 (highlighted in red).

Based on the clinical presentation, patients with MTDPS-5 exhibit similarities to individuals with methylmalonic aciduria, particularly in terms of elevated metabolites. These “atypical” cases of methylmalonic aciduria are likely associated with *SUCLA2* variants. To address this, a more comprehensive genomic testing approach involving single-gene testing, WES, and WGS can be employed. In this study, data on the g.48569263–48571020del1758 variant obtained from WES and WGS were retrospectively compared using IGV visualization software (Supplementary Figure S2). The g.48569263–48571020del1758 variant was not detected by WES due to its location in the 5′ end exon 2, where only a 43 bp region of this variant is present. These findings highlight the potential of WGS in diagnosing diseases that were previously challenging to classify. At present, the MTDPS-5 patient is undergoing symptomatic treatments, including physical therapy to preserve muscle function and prevent joint contractures, antiepileptic drugs, scoliosis and kyphosis bracing, and blepharoplasty. Unfortunately, there are no disease-modifying treatments or established clinical guidelines for managing MTDPS-5. Due to the absence of a definitive diagnosis, the proband has solely undergone physical therapy for muscle function preservation and explored the effectiveness of specific neuro-nutritional medications, which, unfortunately, proved ineffective. For the long-term monitoring of these siblings, coenzyme Q10 supplementation is recommended to improve muscle weakness; however, their parents have declined further specialized care. Subsequently, limited information indicates that PI is currently 9 years old, with no additional details provided. Unfortunately, PII passed away at 1.5 years of age due to respiratory infection.

This clinical case contributes to the understanding of the genotype-phenotype correlation in MTDPS-5 by reporting two novel variants responsible for the condition in Chinese family, thereby raising awareness for this rare disease.

## Data availability statement

The original contributions presented in the study are included in the article/Supplementary material, further inquiries can be directed to the corresponding author.

## Ethics statement

The studies involving humans were approved by the Ethics Committee of Sichuan Academy of Medical Sciences & Sichuan Provincial People’s Hospital. The studies were conducted in accordance with the local legislation and institutional requirements. Written informed consent for participation in this study was provided by the participants’ legal guardians/next of kin. Written informed consent was obtained from the individual(s), and minor(s)’ legal guardian/next of kin, for the publication of any potentially identifiable images or data included in this article.

## Author contributions

XZ: Validation, Methodology, Investigation, Writing – original draft, Conceptualization. GZ: Writing – original draft, Conceptualization. LC: Writing – original draft, Data curation. WZ: Writing – original draft, Validation, Methodology, Investigation. CT: Writing – original draft, Resources. SM: Writing – original draft, Formal analysis. JY: Writing – review & editing, Conceptualization.
