# Supplementary material for: Two novel SUCLA2 variants cause mitochondrial DNA depletion syndrome, type 5 in two siblings
Source: Front Neurol. 2024 Jul 11;15:1394150. doi: 10.3389/fneur.2024.1394150 (PMC11273780; doi:10.3389/fneur.2024.1394150)
Supplement: Supplementary file 1 [file Table_1.docx]

**Supplementary Table S1**. Clinical characteristics in patients with MTDPS-5.

| Patient | Nucleotide change | Predicted amino acid change | Plasma MMA in umol/l (reference values) | Plasma lactate in mmol/l (reference values) | Ethnic origin | Pregnancy and birth | Age at onset/  sex | Presenting symptom(s) | Neurological symptoms | Muscle | Hearing impairment | Other findings | Neuroimaging | Outcome |
| --- | --- | --- | --- | --- | --- | --- | --- | --- | --- | --- | --- | --- | --- | --- |
| PI | Allele 1:c.1234C>T  Allele 2:g.48569263-48571020del1758insATGA | p.Arg412*/? | 7.6  (<4.0) | 3.8  (0.7-2.1) | Chinese | Uncomplicated pregnancy, term birth, BW 2825 g, BL 49 cm | 4mo /M | Hypotonia, feeding diff.  failure to thrive | psychomotor retardation, lack of voluntary movement | Atrophy, contractures, rhabdomyolysis | + | Feeding problems, lack of voluntary movement | MRI: basal ganglia hyperintensities, cerebral atrophy | Alive at 8 y |
| PII | Allele 1:c.1234C>T  Allele 2:g.48569263-48571020del1758insATGA | p.Arg412*/? | 6.9  (<4.0) | 8.6  (0.7-2.1) | Chinese | Uncomplicated pregnancy, term birth, BW 3017 g, BL 51 cm | 5mo /M | Hypotonia | Dystonia, psychomotor retardation | Hypotonia, no head control | + | ? | ? | Alive at 5 mo |
| 1 | Allele 1:c.83delC  Allele 2:c.83delC | p.Asp28vfs*32  p.Asp28vfs*32 | 262  (<130) | 5（<2） | ? | Uncomplicated pregnancy, term birth,BW2850 g, BL 48.5 cm | 5mo /M | Hypotonia,  failure to thrive | Dystonia, psychomotor retardation | Hypotonia, no head control | + | Lack of voluntary movement | MRI: basal ganglia hyperintensities | Alive at 25 mo |
| 2 | Allele 1:Deletion of exon 6  Allele 2:Deletion of exon 6 | p.Tyr222Lysfs*51  p.Tyr222Lysfs*51 | 47（<5） | 4.4（<2） | ? | ? | 2mo /M | Hypotonia,  failure to thrive | Cerebral atrophy | Hypotonia, | + | Feeding problems | Sensorineural hearing loss | Alive at 22 mo |
| 3 | Allele 1:c.998A>G  Allele 2:c.998A>G | p.Asp333Gly  p.Asp333Gly | 2.2 – 3.9 (<0.34) | 0.7  (0.5 –1.7) | Swedish | Preeclampsia, mild asphyxia, BW 2840 g, BL 49 cm | 9 mo/M | FTT | Hypotonia, psychomotor retardation, dystonia, hyperkinesias | Atrophy | - | Feeding problems, reflux, postnatalgrowth retardationowth retardation, decreased spontaneous movement | ? | Alive at 31 y |
| 4 | Allele 1:c.998A>G  Allele 2:c.998A>G | p.Asp333Gly  p.Asp333Gly | 1.8 – 4.0 (<0.34) | 1.4  (0.5 –1.7) | Swedish | Uncomplicated pregnancy, Caesarean section in wk 40, BW 3400 g, BL 51 cm | 3 mo/M | Hypotonia, feeding diff. | Hypotonia, dystonia, psychomotor retardation, choreoathetosis, | Atrophy, contractures, rhabdomyolysis | - | FTT, feeding problems, gastrostomy tube, lack of voluntary movement, reflux, abnormal breathing, hyperhidrosis, postnatalgrowth retardationowth retardation, acute deterioration | CT (1y): normal | Died at 21 y |
| 5 | Allele 1:c.998A>G  Allele 2:c.998A>G | p.Asp333Gly  p.Asp333Gly | 2.9 – 3.2 (<0.4) | 2.5 – 4.5 (<2.3) | Swedish | Uncomplicated pregnancy, term birth, BW 3025 g, BL 49 cm | 5 mo /F | Developmental retardation/arrest | Hypotonia, psychomotor retardation, hyperkinesia | Atrophy | + | FTT, feeding problems, dysarthria | MRI: basal ganglia hyperintensities, leukoencephalopathy, cerebral atrophy | Alive at 27 y: moderate mental retardation, muscle weakness, muscular atrophy, walks w/o support, balance and coordination problems |
| 6 | Allele 1:c.998A>G  Allele 2:c.998A>G | **p.Asp333Gly**  **p.Asp333Gly** | N.d. | 2.1  ( 0.5 –2.2) | ? | Term, BW 4620 g, BL 53 cm | 5 mo/M | Slowgrowth retardationowth retardationowth of HC. athethosis, delayed motor development | Dystonia, ataxia, neuropathy | ? | + | ? | MRI: basal ganglia atrophy | Alive at 7 y |
| 7 | Allele 1:c.998A>G  Allele 2:**Whole gene deletion, 1.54 Mb** | **p.Asp333Gly**  **No protein** | N.d. | 4.6  ( 0.5 –2.2) | ? | Term, BW 3780 g, BL 53 cm | Birth, M | Hypotonia | Athetosis, epilepsy, dystonia, neuropathy | ? | + | ? | MRI: basal ganglia hyperintensities | Alive at 18 y |
| 8 | Allele 1:c.998A>G  Allele 2:c.998A>G | p.Asp333Gly  p.Asp333Gly | N.d. | 3.9 – 12.0 (0.33-1.33) | Finnish | Uncomplicated pregnancy, term birth, BW 4240 g, BL 51 cm | 4 mo/F | Hypotonia, hyperkinesia | psychomotor retardation, lack of voluntary movement | ? | ? | FTT, feeding probl, gastrostomy tube, acute deterioration, slight postnatalgrowth retardationowth retardation, abnormal breathing | MRI (8mo): cerebral atrophy, hemosiderine along wall of left ventricle | Alive at 10 mo: hypotonia, psychomotor retardation, few voluntary movements |
| 9 | Allele 1:c.1219C>T  Allele 2:c.1219C>T | p.Arg407Trp  p.Arg407Trp | 228 (<104) | 4.6  (0.63-2.44) | Caucasian | Caesarean section, BW 2170 g | 3 mo/F | HI | Hypotonia, psychomotor retardation, dystonia, choreoathetosis | ? | + | Neonatal hypoglycemia, loss of speech | MRI (16mo): basal ganglia hyperintensities  MRI (26mo) : basal ganglia lesions, mild cortical atrophy | Alive at 3.5 y |
| 10 | Allele 1:c.1219C>T  Allele 2:c.1219C>T | p.Arg407Trp  p.Arg407Trp | 178.5 -265.2 (<104) | 4.8  (0.63-2.44) | Caucasian | Caesarean section, BW 2310 g | 3 mo/F | HI | Hypotonia, psychomotor retardation, dystonia, choreoathetosis | ? | + | Neonatal hypoglycemia  loss of speech | MRI (16mo): basal ganglia hyperintensities  MRI (26mo) : basal ganglia lesions, mild cortical atrophy | Alive at 3.5 y |
| 11 | Allele 1:c.1219C>T  Allele 2:c.1219C>T | p.Arg407Trp  p.Arg407Trp | N.d. | 4.5 | Pakistani | Uncomplicated pregnancy, Caesarean section due to fetal distress | Birth/M | Feeding problems, recurrent vomiting, HI | Hypotonia, dystonia, psychomotor retardation, hyperkinesias/choreoathetosis, epilepsy | ? | + | FTT, gastrostomy tube, reflux, episodes of acute deterioration, postnatalgrowth retardationowth retardation, no speech | MRI: basal ganglia hyperintensities | Alive age 9 y, constant choreoathetosis |
| 12 | Allele 1:c.1219C>T  Allele 2:c.1219C>T | p.Arg407Trp  p.Arg407Trp | Elevated | 5.7 | Lebanese | Uncomplicated pregnancy, term birth, | 2y/F | Myoclonic-dystonic movement disorder | Dystonia, and myoclonus | myoclonus | + | Communicates with signs and gestures | MRI:putamenal hyperintensity | Died at 34y |
| 13 | Allele 1:c.1219C>T  Allele 2:c.1219C>T | p.Arg407Trp  p.Arg407Trp | Elevated | N.d. | Lebanese | Uncomplicated pregnancy, term birth, | 2y/F | Myoclonic-dystonic movement disorder | Dystonia, and myoclonus | myoclonus | + | Communicates with signs and gestures | MRI:putamenal hyperintensity | Died at 27y |
| 14 | Allele 1:c.1219C>T  Allele 2:c.1219C>T | p.Arg407Trp  p.Arg407Trp | Elevated | N.d. | Lebanese | Uncomplicated pregnancy, term birth, | 2y/M | Myoclonic-dystonic movement disorder | Dystonia, and myoclonus | myoclonus | + | Communicates with signs and gestures | Hyperintensities in basal ganglia with symmetric putamenal invlvement | Died at 23y |
| 15 | Allele 1:Whole gene deletion ~ 258 kb  Allele 2:Whole gene deletion ~ 258 kb | No protein  No protein | N.d. | 4.3 | Pakistani | Uncomplicated pregnancy, cesarean section due to bradycardia in wk 42,dysmature, BW 2570 g, BL 48.5 cm | Birth/F | Contractures, myoclonus, hypotonia, hyper-/hypoglycemia | Hypotonia, psychomotor retardation, dystonia, myoclonus | Contractures, atrophy | ? | Hypoglycemia, FTT, feeding problems, gastrostomy tube | MRI: basal ganglia hyperintensities, cerebral atrophy | Died at 20 y |
| 16 | Allele 1:c.1106dupA  Allele 2:~ 46 kb deletion encompassing exons 1-5 | p.Val370Glyfs*16  No protein | 11.3 (<0.3) | 4.3  (0.3 – 1.1) | Caucasian | Uncomplicated pregnancy, term birth, BW 3335 g, term, BL 49 cm | 2 mo /M | Feeding/swallowing problems | Hypotonia, psychomotor retardation, areflexia, hyperkinesia | Atrophy | + | Feeding problems, gastrostomy tube, recurrent vomiting, reflux, postnatalgrowth retardationowth retardation, hip and shoulder dislocation, scoliosis | MRI: basal ganglia hyperintensities, probably slight cerebral atrophy | Stable at 7 y, severe psychomotor retardation |
| 17 | Allele 1:c.1106dupA  Allele 2:~ 46 kb deletion encompassing exons 1-5 | p.Val370Glyfs*16  No protein | 2.83 (<0.3) | 4.2  (0.3 – 1.1) | Caucasian | Uncomplicated pregnancy, cesarean section at term due to breech pres, BW 3330 g | Birth /M | Feeding/swallowing problems | Hypotonia, dystonia, areflexia, hyperkinesia, psychomotor retardation, epilepsy, reduced nerve conducation velocity | Atrophy | + | Feeding problems, gastrostomy tube, a lot of mucous in airways, postnatalgrowth retardationowth retardation | MRI: basal ganglia hyperintensities, cerebral atrophy | Died at 4 y |
| 18 | Allele 1:c.920C>T  Allele 2:c.920C>T | Creation of an alternative splice donor site resulting in a frameshift | 7.5 – 8.6 (<0.32) | 3.8 | Cape Verde | Medication against preterm birth until birth in wk 33, BW 2155 g | Birth/M | Hypotonia, pneumonia, vomiting, meningitis | Axial hypotonia, hypertonia, hyperkinesia, dystonia, psychomotor retardation, epilepsy | Contractures | + | Feeding problems, gastrostomy tube, vomiting, lack of voluntary mov, reflux, abnormal breathing, tachypnea, postnatalgrowth retardationowth retardation, upslanting palpebral fissures | MRI: basal ganglia hyperintensities, leukoencephalopathy, cerebral atrophy | Stable at 4 y 7 mo |
| 19 | Allele 1:c.750C>A  Allele 2:c.750C>A | p.Tyr250*  p.Tyr250* | 8.4  (<0.4) | 3.8 | Turkish | Polyhydramnios, birth in wk 36, BW 3060 g | Birth/M | Feeding problems, cyanosis | Hypotonia, psychomotor retardation, areflexia | ? | + | FTT, gastrostomy tube, reflux, lack of voluntary movement, postnatalgrowth retardationowth retardation, dysmorphism | MRI (1y): diffuse cerebral atrophy CT (2y): thalamic hypodensities | Died at 2 y due to cerebral infarction and subarachn hemorrhage |
| 20 | Allele 1:**c.308C>A**  Allele 2:**c.308C>A** | **p.Ala103Asp**  **p.Ala103Asp** | N.d. | 3.1  (<2.0) | Caucasian | Pregnancy and birth normal. | 2 mo/F | Hypotonia, FTT, poor weight gain, vomiting | Hypertonia, decreased movements, brisk reflexes, dystonia, tetraparesis, cognitive impairment | Contractures, atrophy | + | Dysphagia, tube feeding, scoliosis | MRI: basal ganglia hyperintensities | Died at 15 y |
| 21 | Allele 1:**c.308C>A**  Allele 2:**c.308C>A** | **p.Ala103Asp**  **p.Ala103Asp** | N.d. | 3.5  (<2.0) | Caucasian | Pregnancy and birth normal | Birth/F | Hypotonia | Hypertonia, dystonia, psychomotor retardation, tetraparesis | ? | + | growth retardation, vomiting, tube feeding, scoliosis, irritability | MRI: basal ganglia hyperintensities, strophy of cerebellum and medulla oblongata | Alive at 7 y |
| 22 | Allele 1:c.1204delA  Allele 2:c.308C>A | p.Ile402Tyrfs*18  p.Ala103Asp | 17 (<0.33) | 2; 4.4 (<2) | Caucasian | Uncomplicated pregnancy, birth in wk 39, BW 2990 g | 6 y/M | Feeding problems, severe hypotonia, acidosis, hyperlactacidemia | Hypotonia,  psychomotor retardation | ? | + | FTT, gastrostomy tube, frequent respiratory infections, tracheostomy, facial dysmorphism, scoliosis | MRI (7 mo): cerebral atrophy, basal ganglia hyperintensities.  MRI (3y ) : leukodystrophy, frontal and temporal atrophy | Alive at 7 y |
| 23 | Allele 1:c.1048G>A  Allele 2:c.1049G>T | p.Gly350Ser  p.Gly350Val | 102 | 5.2 | Japanese/Peruvian | Uncomplicated pregnancy, birth in wk 39, BW 3000 g, BL 49 cm | 3 mo/F | Hypotonia, choreiform movements | Hypertonia, dystonia, psychomotor retardation, hyperkinesias, mild spasticity | Atrophy | + | FTT, feeding problems, hyperhidrosis, postnatalgrowth retardationowth retardation | MRI (1y and 2y): cerebral atrophy | Alive at 5 y 10 mo |
| 24 | Allele 1:c.1048G>A  Allele 2:c.1049G>T | **p.Gly350Ser**  **p.Gly350Val** | N.d. | Elevated | ? | ? | 4 mo/F | Delayed motor development, FTT, hypotonia, dyskinesia | ? | ? | + | ? | MRI: mild cerebral atrophy | Alive at 14 mo |
| 25 | Allele 1:c.1271delG  Allele 2:c.1271delG | p.Gly424Aspfs*18  p.Gly424Aspfs*18 | N.d. | 4.6 | Pakistani | Caesarean section due to breech position in wk 39, BW 4100 g, Apgar scores 9/1, 9/5 | Birth/M | HI, hypotonia | Dystonia, hyperkinesias, psychomotor retardation, areflexia | ? | + | FTT, feeding problems, kyphoscoliosis, no speech | MRI: basal ganglia hyperintensities, atrophy | Alive at 5.5 y |
| 26 | Allele 1:**c.985A>G**  Allele 2:**c.985A>G** | **p.Met329Val**  **p.Met329Val** | + | 5.1 (0.5–2.2) | ? | ? | 6mo/M | Hypotonia, vomiting | psychomotor retardation, microcephaly, dystonia | Atrophy | + | growth retardation, FTT, facial dysmorphism | ? | Alive at 14 y |
| 27 | Allele 1:**c.985A>G**  Allele 2:**c.985A>G** | **p.Met329Val**  **p.Met329Val** | N.d. | 2.2 – 9.0 (<1.3) | ? | Uncomplicated pregnancy and birth. BW 3000g, BL 50 cm, | Birth/M | Hypotonia, vomiting | psychomotor retardation, microcephaly, dystonia | Atrophy | + | growth retardation, FTT, facial dysmorphism | MRI: cerebral atrophy, basal ganglia hypointensities | Alive at 2 y |
| 28 | Allele 1:**c.751G>A**  Allele 2:**c.751G>A** | **p.Asp251Asn**  **p.Asp251Asn** | N.d. | 23.8  (4.5 -20.0) | Iran | Cesarean section, BW 2900 g | 1 y/F | Delayed motor development, FTT, hearing loss | Hypotonia, dystonia, encephalopathy | ? | + | Feeding problems, FTT | MRI: basal ganglia hyperintensities | Alive at 4 y |
| 29 | Allele 1:**c.751G>A**  Allele 2:**c.751G>A** | **p.Asp251Asn**  **p.Asp251Asn** | N.d. | N.d. | Iran | Cesarean section, BW 2700 g | 2 y/F | Hearing loss | Dystonia, gait problems | ? | + | Dysarthria, dyphagia | MRI: basal ganglia hyperintensities | Alive at 10 y |
| 30 | Allele 1:**c.789_802+29delinsATAAA**  Allele 2:**c.789_802+29delinsATAAA** | **p.Asp263_Ile463delinsGlu**  **p.Asp263_Ile463delinsGlu** | N.d. | 4.3  (<2.1) | Muslim | BW, 2250 g (<3^rd^ percentile) | 1 mo/F | Irritability | Hypotonia, psychomotor retardation, seizures, dystonia | Contractures | + | Frequent respiratory infections, GI reflux, anemia | MRI: basal ganglia hyperintensities, atrophy | Alive at 7 y |
| 31 | Allele 1:**c.789_802+29delinsATAAA**  Allele 2:**c.789_802+29delinsATAAA** | **p.Asp263_Ile463delinsGlu**  **p.Asp263_Ile463delinsGlu** | N.d. | 4.0 – 5.0 (< 2.1) | Muslim | BW, 2180 g, <3rd percentile | 2 mo/M | Unresponsive, hypopneic | Hypotonia, lack of voluntary movements, seizures | ? | + | Anemia | CT: basal ganglia infarcts | Alive at 5 y |
| 32 | Allele 1:**c.850C>T**  Allele 2:**c.850C>T** | **p.Arg284Cys**  **p.Arg284Cys** | 1.3 (<0.33) | 4.0-6.0 (0.5-1.7) | Italian | Term, BW 3500 g | 3 mo/M | Motor retardation, hypotonia | Delayed motor development, hypotonia, dystonia, psychomotor retardation, | ? | + | Feeding problems, GI reflux | Cerebral atrophy, basal ganglia involvement | Alive at 2 y |
| 33 | Allele 1:**c.850C>T**  Allele 2:**c.850C>T** | **p.Arg284Cys**  **p.Arg284Cys** | N.d. | 3.0 | Italian | ? | 4 mo/M | ? | Delayed motor development, hypotonia, dystonia, spasticity, psychomotor retardation | ? | + | FTT, feeding problems | Basal ganglia involvement | Alive at 6 y |
| 34 | Allele 1:**c.850C>T**  Allele 2:**c.352G>A** | **p.Arg284Cys**  **p.Gly118Arg** | 2.4 | 7.5 | Italian/Romanian | ? | 4 mo/M | ? | Delayed motor development, hypotonia, dystonia, psychomotor retardation | ? | ? | FTT, feeding problems | Cerebral atrophy, basal ganglia involvement | Alive at 1.2 y |
| 35 | Allele 1:**c.160_161insAGA**  Allele 2:c.850C>T | **p.Ser54***  p.Arg284Cys | N.d. | 1.36 (0.63-2.44) | Caucasian | Uncomplicated pregnancy, birth in wk 39, BW 3440 g, BL 50 cm | 4 mo/M | Hypotonia, feeding problems | Hypotonia, dystonia, psychomotor retardation | Atrophy | + | Feeding problems, recurrent airway infections, deterioration during infections | MRI (5 mo) : cerebral atrophy, hyperintensities in putamen and caudate nucleus | Alive at 1,5 y |
| 36 | Allele 1:c. 534+1G>A  Allele 2:c. 534+1G>A | **Skipping of exon 4**  **Skipping of exon 4** | N.d. | N.d. | Faroese | ? | Birth/F | ? | Delayed motor development, hypotonia | ? | + | ? | ? | Died at 0.8 y |
| 37 | Allele 1:c. 534+1G>A  Allele 2:c. 534+1G>A | **Skipping of exon 4**  **Skipping of exon 4** | N.d. | N.d. | Faroese | ? | ? | ? | Hypotonia | ? | ? | ? | ? | Died at 0.3 y |
| 38 | Allele 1:c. 534+1G>A  Allele 2:c. 534+1G>A | **Skipping of exon 4**  **Skipping of exon 4** | N.d. | N.d. | Faroese | ? | ? | ? | Delayed motor development, hypotonia, epilepsy, dystonia, spasticity, psychomotor retardation | ? | + | FTT, feeding problems, | Cerebral and cerebellar atrophy | Died at 45 y |
| 39 | Allele 1:c. 534+1G>A  Allele 2:c. 534+1G>A | **Skipping of exon 4**  **Skipping of exon 4** | N.d. | N.d. | Faroese | ? | 6 mo/F | ? | Delayed motor development, hypotonia, dystonia, spasticity, | ? | + | FTT, feeding problems, scoliosis, pulmonary infections, vomiting | ? | Alive at 7 y |
| 40 | Allele 1:c. 534+1G>A  Allele 2:c.985A>G | Skipping of exon 4  p.Met329Val | 2.4  (<0.5) | 6.3  (0.4 –2.0) | Caucasian | Uncomplicated pregnancy, birth wk 41, BW 3100 g, BL 49.5 cm | Birth/F | HI | Hypotonia, dystonia, psychomotor retardation | Atrophy | + | Sleep disturbances | MRI: basal ganglia hyperintensities | Alive at 35 mo: hypotonia, constant but slow improvement |
| 41 | Allele 1:c. 534+1G>A  Allele 2:c.985A>G | Skipping of exon 4  p.Met329Val | 924  (73-271) | 48  (4.5–19.8) | Caucasian | ? | Birth/F | HI | psychomotor retardation | ? | + | FTT, feeding problems | MRI: possible choroid plexus cyst, patchy areas of subcortical white matter hyperintensity | Alive at 4 y |
| 42 | Allele 1:c. 534+1G>A  Allele 2:c. 534+1G>A | Skipping of exon 4  Skipping of exon 4 | N.d. | N.d. | Faroese | Uncomplicated pregnancy, birth in wk 39, BW 3875 g, BL 51 cm | Birth/F | Hypotonia, feeding problems | Hypotonia, psychomotor retardation | Hypotonia, no head control | + | Gastrostomy tube, no antigrowth retardationavity movements, cochlear implant | ? | Alive at 3 y |
| 43 | Allele 1:c. 534+1G>A  Allele 1:c. 534+1G>A | Skipping of exon 4  Skipping of exon 4 | 20  (<0.33) | 5.9  (<2.0) | Caucasian | Preterm birth in wk 36, BW 2174 g, Apgar scores 1/5, 5/8 | Birth/M | Feeding problems, respiratory distress, severe jaundice | Hypotonia, psychomotor retardation, EEG abnormalities | ? | + | Mild facial dysmorphism | MRI (2y): leukoencephalopathy | Alive at 8 y |
| 44 | Allele 1:c. 534+1G>A  Allele 2:c. 534+1G>A | **Skipping of exon 4**  **Skipping of exon 4** | 2.4 (<0.28) | 1.9-3.0 (<2.0) | Faroese | Cesarean section, 3055 g, 50 cm | 1 mo/M | Hypotonia, muscular atrophy, FTT | Dystonia, psychomotor retardation | Atrophy | + | growth retardation | MRI: basal ganglia hyperintensities, atrophy | Alive 10 y |
| 45 | Allele 1:c. 534+1G>A  Allele 2:c. 534+1G>A | **Skipping of exon 4**  **Skipping of exon 4** | 2.5 | 8.6  (<2.0) | Faroese | Cesarean section, 2-3 wks post term, 3500 g, 54 cm | 2.5 mo/F | Hypotonia, fatigue | Dystonia, psychomotor retardation | Atrophy | + | Hyperhidrosis,growth retardationowth retardation | ? | Alive 16 y |
| 46 | Allele 1:c. 534+1G>A  Allele 2:c. 534+1G>A | **Skipping of exon 4**  **Skipping of exon 4** | 2.7 | 5.4-5.9 (<2.0) | Faroese | Dysmature, 2800 g, 49 cm | 3.5mo/M | Motor retardation, hypotonia | Dystonia,psychomotor retardation | Atrophy | + | Hyperhidrosis,growth retardationowth retardation | CT: central and cortical atrophy | Died 21 y |
| 47 | Allele 1:c. 534+1G>A  Allele 2:c. 534+1G>A | **Skipping of exon 4**  **Skipping of exon 4** | N.d. | 0.9-1.4 (<2.0) | Faroese | Dysmature, 2900 g, 53 cm | 5 mo/M | Motor retardation | Dystonia, psychomotor retardation | Atrophy | + | Hyperhidrosis,growth retardationowth retardation | ? | Died 10 y |
| 48 | Allele 1:c. 534+1G>A  Allele 2:c. 534+1G>A | **Skipping of exon 4**  **Skipping of exon 4** | N.d. | N.d. | Faroese | Oligohydramnios, dysmature | 2 mo/M | Hypotonia, hyperhidrosis, hearing impairment | Dystonia, psychomotor retardation | Atrophy | + | growth retardation | MRI: central and cortical atrophy | Alive 6 y |
| 49 | Allele 1:c. 534+1G>A  Allele 2:c. 534+1G>A | **Skipping of exon 4**  **Skipping of exon 4** | N.d. | N.d. | Faroese | Pregnancy and birth uncomplicated | 4 mo/M | Hypotonia | Dystonia, psychomotor retardation | Atrophy | + | growth retardation | CT: basal ganglia atrophy, generalised atrophy | Died 15 y |
| 50 | Allele 1:c. 534+1G>A  Allele 2:c. 534+1G>A | **Skipping of exon 4**  **Skipping of exon 4** | 0.8 | 4.9 (<2.0) | Faroese | Pregnancy and birth uncomplicated, 3750 g | 5 mo/F | Hypotonia | Dystonia, psychomotor retardation | Atrophy | + | growth retardation | MRI: slight atrophy | Alive 2 y |
| 51 | Allele 1:c. 534+1G>A  Allele 2:c. 534+1G>A | **Skipping of exon 4**  **Skipping of exon 4** | N.d. | N.d. | Faroese | Pregnancy and birth uncomplicated, 3650 g, 53 cm | 3 mo/F | Hypotonia | Dystonia, psychomotor retardation | Atrophy | + | growth retardation | ? | Died 10 y |
| 52 | Allele 1:c. 534+1G>A  Allele 2:c. 534+1G>A | **Skipping of exon 4**  **Skipping of exon 4** | N.d. | N.d. | Faroese | Normal pregnancy and birth, 3400 g, 53 cm | Birth/M | Hypotonia | Dystonia, psychomotor retardation | Atrophy | + | ? | CT: basal ganglia atrophy, amterior horn atrophy | Died 18 y |
| 53 | Allele 1:c. 534+1G>A  Allele 2:c. 534+1G>A | **Skipping of exon 4**  **Skipping of exon 4** | N.d. | 3.7-11.4 (<2.0) | Faroese | Normal pregnancy and birth | Birth/M | Infantile spasms | Dystonia, psychomotor retardation | Atrophy | + | Hyperhidrosis,growth retardationowth retardation | MRI: atrophy, delayed myelination | Died 4 y |
| 54 | Allele 1:c. 534+1G>A  Allele 2:c. 534+1G>A | **Skipping of exon 4**  **Skipping of exon 4** | N.d. | 2.1 (<2.0) | Faroese | Normal pregnancy and birth, 3905 g, 55 cm | Birth/M | Hypotonia | Dystonia, psychomotor retardation | Atrophy | ? | ? | ? | Died 8 mo |
| 55 | Allele 1:c. 534+1G>A  Allele 2:c. 534+1G>A | **Skipping of exon 4**  **Skipping of exon 4** | 33.0 | 5.4-5.6 (<2.0) | Faroese | Twin birth,38 wks, 2410 g, 48 cm | Birth/F | Hypotonia | Dystonia, psychomotor retardation | Atrophy | + | growth retardation | MRI: basal ganglia hyperintensities, atrophy | Alive 5 y |
| 56 | Allele 1:c. 534+1G>A  Allele 2:c. 534+1G>A | **Skipping of exon 4**  **Skipping of exon 4** | N.d. | N.d. | Faroese | ? | 3 mo/M | FTT, weak suck | Delayed motor development, hypotonia, microcephaly | ? | + | Feeding problems, tracheomalacia | Cerebral atrophy | Alive 2.5 y |

MMA: methylmalonic acid,N: normal, M: male, F: female, FTT: failure to thrive, HI: hearing impairment, GI: gastrointestinal, MRI: magnetic resonance imaging, N.d.: not done,

?: unknown, +: present, -: absent, wks: weeks, mo: month(s), y: year(s), BW: birth weight, BL: birth length
