# Supplementary material for: Two novel SUCLA2 variants cause mitochondrial DNA depletion syndrome, type 5 in two siblings
Source: Front Neurol. 2024 Jul 11;15:1394150. doi: 10.3389/fneur.2024.1394150 (PMC11273780; doi:10.3389/fneur.2024.1394150)
Supplement: Supplementary file 2 [file Data_Sheet_1.docx]

**Supplementary Figure 1** In silico analysis. Functional analysis of SUCLA2 variants in the protein structure was performed using PyMOL software. **A.** WT: The three-dimensional structure of the wild type (blue); **B.** MT-1: mutant *SUCLA2* (g.48569263–48571020del1758insATGA variant) gene-encoded protein (mt: light blue; wt: pink); **C.** MT-2: mutant *SUCLA2* (c.1234C>T variant) gene-encoded protein (mt: green; wt: pink). Enlargements of regions that contained the affected amino acids are displayed.


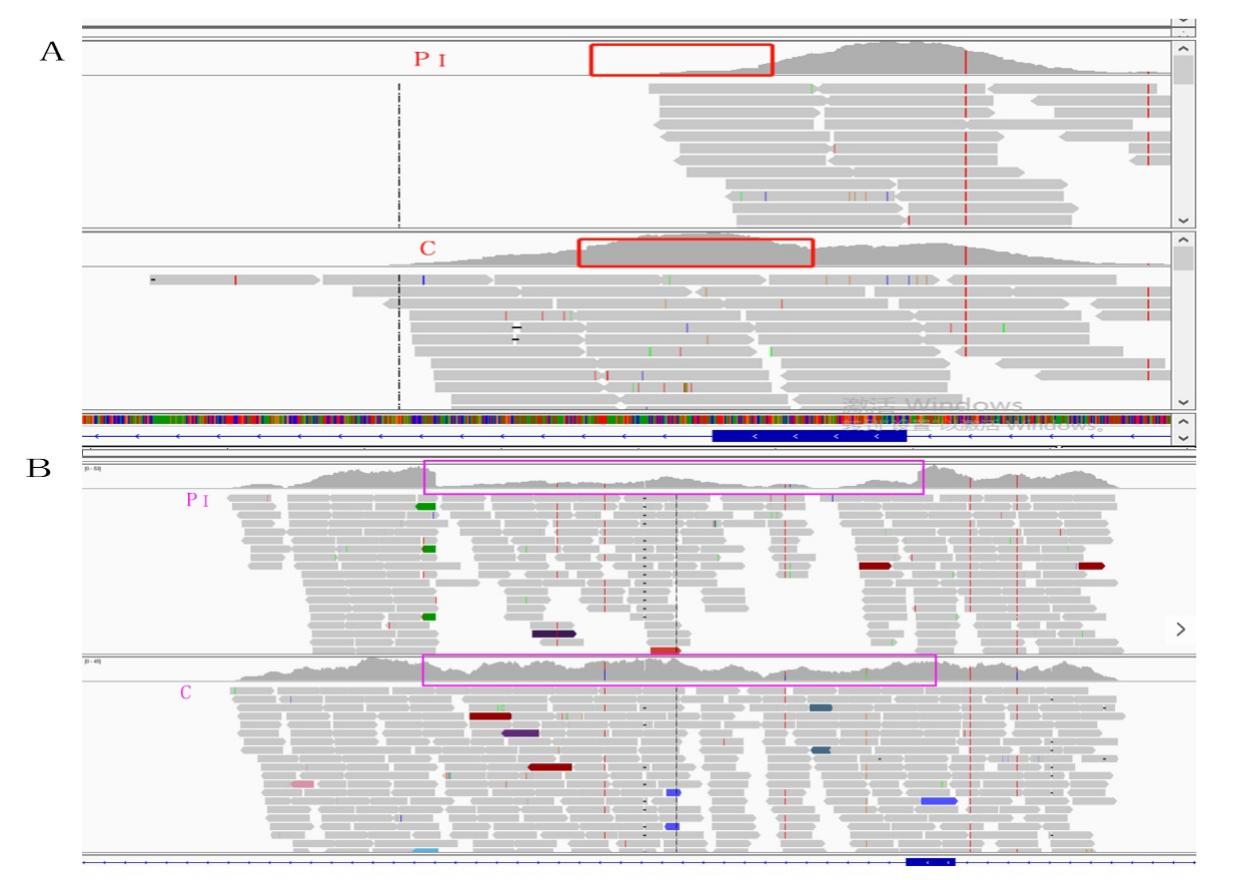


**Supplementary Figure 2** The g.48569263-48571020del1758 variant data obtained from the WES and WGS were retrospectively compared via IGV visualization software. The g. 48569263-48571020del1758 location in WES was compared to that in the control (red frame); the g.48569263-48571020del1758 location in WGS was compared to that in the control (pink frame).
